# Cure Kinetics-Driven Compression Molding of CFRP for Fast and Low-Cost Manufacturing

**DOI:** 10.3390/polym17152154

**Published:** 2025-08-06

**Authors:** Xintong Wu, Ming Zhang, Zhongling Liu, Xin Fu, Haonan Liu, Yuchen Zhang, Xiaobo Yang

**Affiliations:** 1 School of Advanced Manufacturing, Nanchang University, Nanchang 330031, China; 13117806093@163.com (M.Z.);; 2 Nanchang Jardine Advanced Composite Material Co., Ltd., Nanchang 330029, China; 3 College of Mechanical and Electrical Engineering, Lanzhou Jiaotong University, Lanzhou 730070, China; 4 State Key Laboratory of Precision Manufacturing for Extreme Service Performance, Central South University, Changsha 410083, China

**Keywords:** carbon fiber-reinforced polymer composites, curing kinetics, curing cycle optimization, differential scanning calorimetry

## Abstract

Carbon fiber-reinforced polymer (CFRP) composites are widely used in aerospace due to their excellent strength-to-weight ratio and tailorable properties. However, these properties critically depend on the CFRP curing cycle. The commonly adopted manufacturer-recommended curing cycle (MRCC), designed to accommodate the most conservative conditions, involves prolonged curing times and high energy consumption. To overcome these limitations, this study proposes an efficient and adaptable method to determine the optimal curing cycle. The effects of varying heating rates on resin dynamic and isothermal–exothermic behavior were characterized via reaction kinetics analysis using differential scanning calorimetry (DSC) and rheological measurements. The activation energy of the reaction system was substituted into the modified Sun–Gang model, and the parameters were estimated using a particle swarm optimization algorithm. Based on the curing kinetic behavior of the resin, CFRP compression molding process orthogonal experiments were conducted. A weighted scoring system incorporating strength, energy consumption, and cycle time enabled multidimensional evaluation of optimized solutions. Applying this curing cycle optimization method to a commercial epoxy resin increased efficiency by 247.22% and reduced energy consumption by 35.7% while meeting general product performance requirements. These results confirm the method’s reliability and its significance for improving production efficiency.

## 1. Introduction

CFRP composites have become irreplaceable structural materials in aerospace and high-end manufacturing due to their exceptional specific strength, corrosion resistance, and customizable mechanical properties [1,2,3,4]. Their customizable mechanical properties can meet the various requirements for material performance in different high-end manufacturing scenarios. However, to fully realize the potential of this material, an appropriate and efficient curing cycle is an essential prerequisite. The industry currently relies predominantly on the MRCC as the standard protocol [5]. Despite its widespread adoption, the MRCC is severely constrained by its need to accommodate the most conservative scenarios, such as maximum-thickness components, forcing it to employ excessively long curing cycles that severely impede production efficiency [6]. Moreover, the continuous high-temperature and high-pressure conditions of the MRCC result in extremely high energy consumption. This contradicts global green manufacturing goals and hinders sustainable industrial growth. Optimizing the curing cycle is therefore not merely an academic pursuit but a crucial engineering challenge with direct implications for cost competitiveness, production capacity, and sustainability in CFRP manufacturing.

To optimize the curing cycle of epoxy-based CFRP while balancing efficiency, material performance, and energy conservation, researchers have pursued two primary pathways through extensive investigation. For the first approach, some researchers focus on resin formulation design to accelerate curing through chemical composition modifications. Eom et al. [7] investigated the impact of various curing agents on the curing behavior of epoxy resins to reduce curing time and improve efficiency. Meanwhile, Tarafdar et al. [8] employed an improved ultraviolet-assisted frontal polymerization (UV-FP) method, utilizing ultraviolet-triggered self-exothermic reactions to complete the curing process. This approach reduced curing time from 15 h to less than 2 min, achieving mechanical properties comparable to or surpassing those of traditional curing methods. However, this method may cause non-uniform temperature distribution due to self-sustained exothermic reactions during the frontal polymerization process, potentially leading to adverse effects. For the second approach, some researchers are dedicated to achieving accelerated curing by coupling external energy fields. For example, Tang et al. [9] achieved highly efficient and energy-saving curing of epoxy resins through electro-curing, reducing energy consumption by 90% and shortening curing time by 40% compared to autoclave processing. Nevertheless, this method significantly increased porosity and substantially decreased mechanical properties, rendering it unsuitable for high-performance applications. Similarly, Xu et al. [10] employed microwave curing to enhance the compressive strength of CFRP composites while reducing curing time by 39%. However, the intricate multiphase dynamics during microwave curing demand further comprehensive study in composite manufacturing. Additionally, Muc et al. [11] described the influence of resin flow and curing dynamics on residual stresses in composite structures and optimized the curing process through numerical analysis and genetic algorithms. Researchers have increasingly employed curing kinetics and rheological models to understand and predict the complex interplay of heat transfer, chemical reactions, and resin flow during curing. The phenomenological Kamal model and autocatalytic models are widely used [12,13,14,15].

Although the aforementioned studies demonstrate potential in specific scenarios, a universal method for CFRP curing cycle optimization remains absent due to the intrinsic complexities of thermoset composites. CFRP components with different geometries each require their own unique optimal curing process parameters to balance the trade-offs between efficiency, performance, and energy consumption. The intrinsic design–manufacturing interdependency of CFRP necessitates that cure parameters (e.g., heating rate, temperature, dwell time) simultaneously satisfy multi-objective constraints: high performance, high efficiency, and low energy consumption. However, conventional parameter determination methods can only characterize curing cycles under isolated conditions, failing to systematically evaluate interactive effects of multivariate combinations on comprehensive outcomes. The urgent need to establish novel methodologies to overcome the limitations of the MRCC has become a critical challenge in CFRP manufacturing.

This study aims to develop an optimized, rapid, and low-energy compression molding cure cycle for epoxy resin prepreg systems. Although the MRCC provides a safe baseline, its inefficiency is particularly pronounced for this widely used material. The core limitation of current practice lies in the reliance on empirical trial-and-error or isolated DSC analysis to formulate cure cycles, lacking a systematic, model-driven approach to efficiently explore the vast process parameter space. This study proposes a dynamic optimization strategy: by elucidating the curing kinetics and rheological behavior of resins and systematically investigating the impact of curing process parameter combinations on mechanical properties through orthogonal experiments, a weighted scoring system encompassing performance, energy consumption, and compression molding time is established to conduct a multidimensional comprehensive evaluation of the MRCC and optimized alternatives. This approach enables the customization of the optimal process route on demand. Compared to the MRCC, the curing cycle routes formulated by this method are more flexible and realistic, effectively reducing unnecessary energy consumption and minimizing wasted compression molding time.

## 2. Materials and Methods

### 2.1. Materials

This study employs T700/2626 prepreg provided by Nanchang Jardine Advanced Composite Material Company, Nanchang, China. It features an epoxy resin system with significant toughening effects. The carbon fibers are PAN-based, are prepared by wet spinning, and exhibit strong adhesion between fiber and matrix. The prepreg has a fiber volume fraction of 67% and a single-layer thickness of approximately 0.125 mm.

### 2.2. DSC Measurements

In this study, the thermal behavior of the epoxy system was investigated using the DSC 3500 Sirius manufactured by NETZSCH, Selb, Bavaria, Germany. An aluminum crucible with a volume of 40 μL was used, and the mass of the resin was controlled to be 5–10 mg. A nitrogen purge flow rate of 40 mL/min was selected, following similar procedures and verified to effectively maintain an inert atmosphere without adversely affecting the specimen [16]. The dynamic experimental temperature range was 300–550 K, with heating rates of 1 K/min, 2 K/min, 3 K/min, 4 K/min, 5 K/min, 10 K/min, 15 K/min, and 20 K/min. In order to investigate the isothermal DSC behavior of the sample under different heating rates, the crucible containing the sample was placed into the furnace and heated to the target temperatures of 393 K, 413 K, and 433 K at various heating rates of 1 K/min, 5 K/min, 10 K/min, and 20 K/min, with the heat flow signal being recorded simultaneously [17]. To guarantee the reliability of the experimental data, the DSC was calibrated using indium and zinc. After each experiment, all sample crucibles were reweighed to detect any possible leakage during the experimental process. Moreover, all DSC tests were conducted in triplicate to verify the reproducibility of the results.

### 2.3. Rheological Measurements

In this study, the rheological properties of thermosetting materials were systematically investigated under both non-isothermal and isothermal conditions using steady-state shear flow measurement techniques [18]. Prior to the experiments, a series of preliminary tests were conducted to optimize the experimental parameters. The final parameters were determined as follows: a gap of 1 mm, an angular frequency of 10 rad/s, and a strain of 1%. Under non-isothermal experimental conditions, the heating rates were set at 1 K/min, 5 K/min, 10 K/min, 15 K/min, and 20 K/min, covering a temperature range of 300–550 K. Under isothermal experimental conditions, the selected temperatures were 373 K, 393 K, 413 K, 433 K, and 453 K. Analysis of the isothermal rheological curves further explores the mechanism of the curing reaction and the key role of viscosity during the curing cycle.

### 2.4. Compression Molding Process

**Figure 1:**
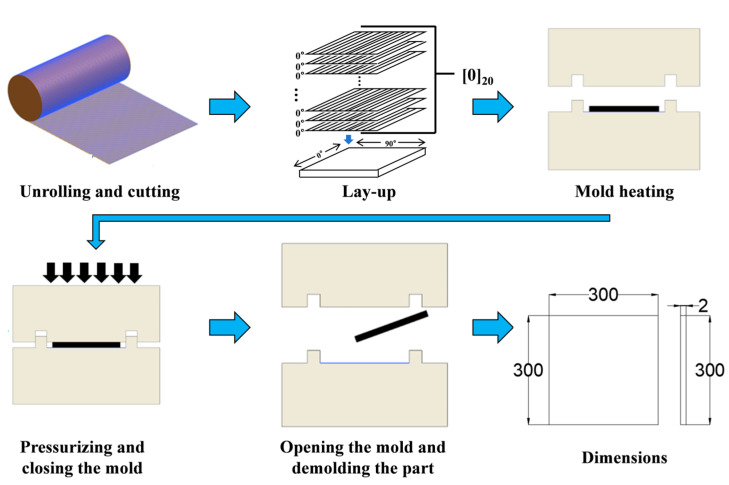
Schematic diagram of compression molding.

This study employs a compression molding technique with manual layup of 20 layers in a [0]_20_ configuration, embedding temperature detection equipment at layers 0, 10, and 20, as shown in Figure 1. The process includes the following steps: First, the carbon fiber/epoxy prepreg is removed from −18 °C storage and thawed at approximately 25 °C for six hours. Once moisture-free, it is cut into 300 mm × 300 mm pieces. Next, the fiber direction is defined as 0°, and 20 layers of prepreg are laid up manually. Each layer is compacted using tools such as rubber rollers or scrapers to ensure tight bonding and remove air bubbles, while temperature sensors are embedded at layers 0, 10, and 20. Subsequently, the laid-up prepreg stack is placed into the mold of a BL-6170-B flat-plate press and the curing program is executed at a forming pressure of 0.6 MPa. Finally, after curing, the mold is cooled to below 60 °C at a rate of 2 K/min. The mold is then opened, and the cured composite laminate sample is removed.

### 2.5. Interlaminar Shear Strength Test

**Figure 2:**
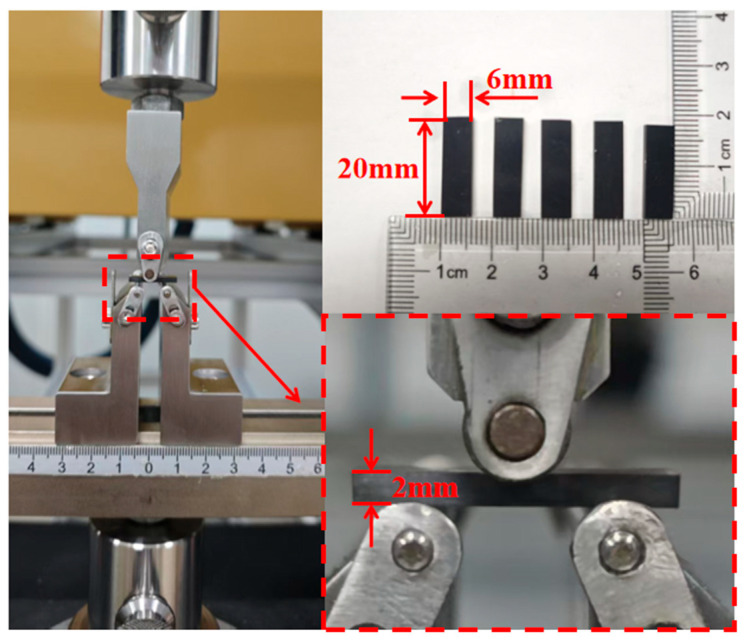
A schematic of the short beam three-point bending test.

Interlaminar shear strength (ILSS) is a critical mechanical test for evaluating the interlaminar bonding performance of composite materials. This experiment assesses the structural integrity and durability of materials by measuring their resistance to shear failure between layers. ILSS exhibits greater sensitivity to the curing degree of CFRP than tensile or flexural properties. Therefore, ILSS is adopted for CFRP evaluation. The short beam shear test was conducted in accordance with GB/T 30969–2014 [19]. The standard specimen dimensions are 20 mm × 6 mm × 2 mm (length × width × thickness), as shown in Figure 2. These specimens were cut from a unidirectional laminate measuring 300 mm × 300 mm, fabricated from 20 layers of T700/2626 prepreg. During the test, a Zwick testing machine applied a loading rate of 1.0 mm/min to the specimen and measured the applied load. The short beam three-point bending strength (interlaminar shear strength, ILSS) was calculated using Equation (1) [20]:

(1) $$\tau_{sbs}=0.75\times \left(\frac{P_{max}}{Wh}\right)$$

where τ_sbs_, P_max_, W, and h represent the shear strength, the maximum load observed during the test, the specimen width, and the specimen thickness, respectively. Metallographic examination was employed to determine the porosity of compression-molded carbon fiber/epoxy composites. The advanced image processing software “image Pro plus 6.0” was used to calculate the pore area ratio in the metallographic image of the polished sample, so as to quantify the porosity parameters [21,22].

## 3. Results

### 3.1. Epoxy Curing Behavior Analysis

#### 3.1.1. Dynamic Curing Kinetics Characterization and Modeling

**Figure 3:**
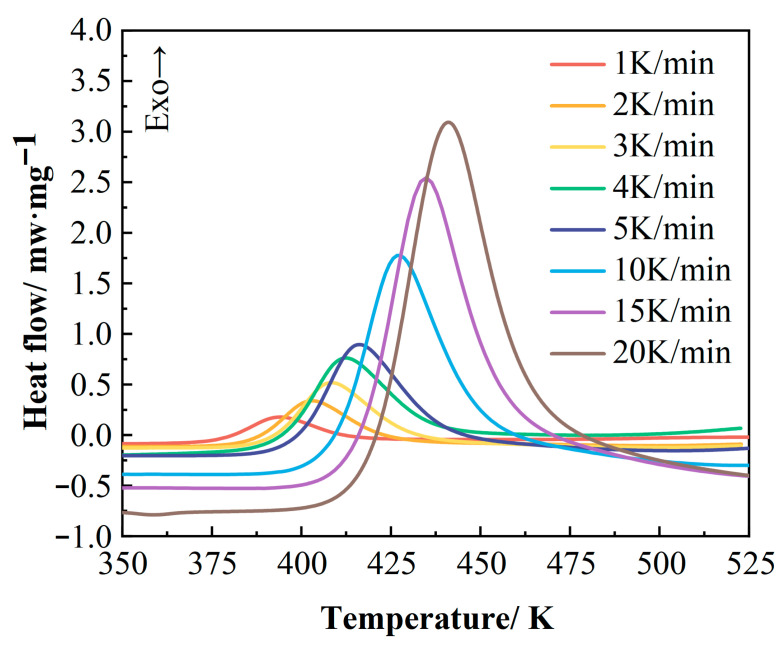
Dynamic DSC curves of epoxy resin at different heating rates.

Thermal analysis was performed using DSC to determine the curing temperature range of the 2626 epoxy resin. Figure 3 presents the DSC curves of this epoxy resin obtained at different heating rates (1, 2, 3, 4, 5, 10, 15, and 20 K/min). From these curves, the onset temperature (T_i_), peak temperature (T_p_), and termination temperature (T_f_) of the curing reaction were determined; specific values are listed in Table 1. The resin exhibits only a single exothermic peak throughout the entire curing process. Furthermore, its characteristic temperatures (T_i_, T_p_, T_f_) increase with higher heating rates, and the corresponding exothermic peak shifts entirely towards higher temperatures [23,24]. This phenomenon is primarily attributed to the increased thermal effect per unit time at higher heating rates, which intensifies thermal lag within the system [25].

**Figure 4:**
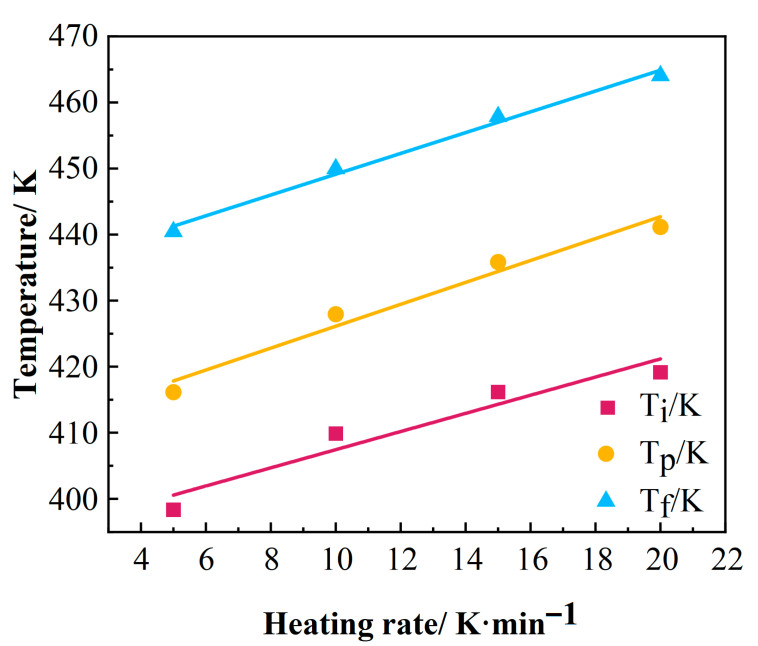
Extrapolation curves of curing temperature for the resin.

To determine the characteristic curing temperatures—onset temperature (T_i_), peak temperature (T_p_), and termination temperature (T_f_)—of the epoxy resin, the characteristic temperature data obtained at different heating rates (β) were subjected to linear regression [26]. These fitted lines were extrapolated to a heating rate of β = 0 K/min, as illustrated in Figure 4. The results indicate a strong linear relationship between the characteristic temperatures and the heating rate. Consequently, the intercept values of each linear regression curve on the y-axis (corresponding to β = 0) represent the extrapolated characteristic temperatures for the curing process under quasi-static conditions [27]. Based on this analysis, the characteristic curing temperatures of the resin were determined as follows: Ti = 393 K, Tp = 413 K, Tf = 433 K.

**Figure 5:**
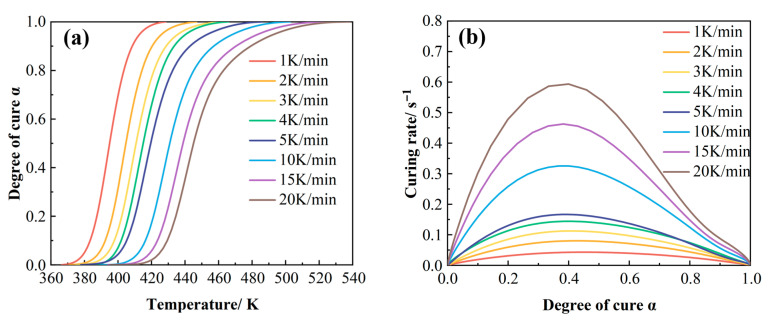
(**a**) Curves of the relationship between degree of cure and temperature at different heating rates; (**b**) curves of the relationship between reaction rate and degree of cure.

The degree of cure as a function of temperature (Figure 5a) was obtained by calculating the peak area of the non-isothermal DSC curves and normalizing the partial peak area at various temperatures with respect to the total peak area according to Equation (2) [28,29].

(2) $$\alpha \left(T\right)=\frac{∆H_{t}}{∆H_{0}}$$

where $\alpha \left(T\right)$ is the curing degree at T, $∆H_{t}$ is the heat release at T, and $∆H_{0}$ is the total heat release of the curing reaction.

The results demonstrate that the temperature required to achieve the same degree of cure increases with higher heating rates [30]. This phenomenon arises from the competition between two opposing effects induced by higher heating rates: (1) a reduction in system viscosity, which facilitates resin flow and void removal, and (2) an acceleration of the curing reaction kinetics, which rapidly increases viscosity and gelation, thereby limiting the time available for resin flow and consolidation [31]. Furthermore, increased heating rates lead to higher onset (T_i_) and peak (T_p_) temperatures, causing the entire curve to shift towards higher temperatures. This phenomenon stems from delayed molecular chain mobility in response to temperature changes, postponing the initiation of crosslinking reactions and consequently requiring higher temperatures to trigger the curing process [32].

The curing reaction rate was obtained by differentiating the degree-of-cure curve with respect to time [33,34]. Figure 5b illustrates the variation in the curing rate as a function of the degree of cure at different heating rates. It can be seen that the curing rate reaches its maximum at a degree of cure of approximately 0.4, subsequently decreasing as the degree of cure increases. This behavior is consistent with the characteristics of an autocatalytic reaction mechanism [35]. Furthermore, at any given degree of cure, a higher heating rate corresponds to a greater curing reaction rate.

**Figure 6:**
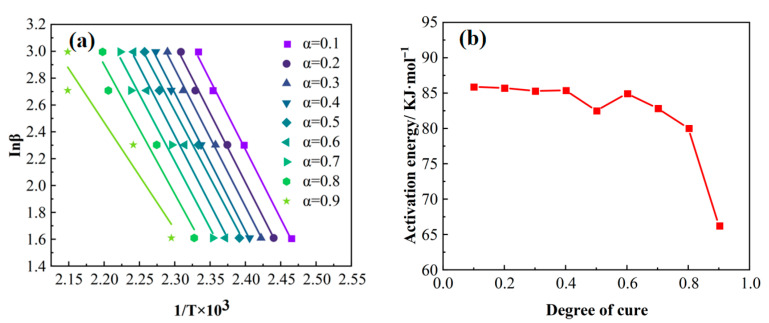
(**a**) FWO method fitting curves for resin at different degrees of cure; (**b**) variation in resin activation energy with degree of cure (FWO method).

The activation energy (Ea) corresponding to different degrees of cure (α) during the curing process was calculated using the Flynn–Wall–Ozawa (FWO) methods [36]. Figure 6 presents the corresponding linear fitting curves and the variation in the activation energy (Ea) as a function of the degree of cure (α). Analysis revealed a significant change in activation energy when the degree of cure exceeded 0.4. Based on this characteristic variation in Ea, a variable activation energy model—specifically, the modified Sun–Gang model—was selected to describe the curing kinetics of this epoxy resin, whose general form is given by Equation (3) [37].

(3) $$\beta \frac{d\alpha }{dT}=exp\left(p_{1}+p_{2}\alpha +p_{3}\alpha^{2}+p_{4}\alpha^{3}\right)exp\left(-\frac{p_{5}+p_{6}\alpha +p_{7}\alpha^{2}+p_{8}\alpha^{3}}{RT}\right)\alpha^{m}{\left(1-\alpha \right)}^{n}$$

where β is the heating rate, $\frac{d\alpha }{dT}$ is the curing rate, P_1_–P_8_ is the model parameter, R is the universal gas constant, *m* and *n* are the reaction order, and T is the thermodynamic temperature.

Using the degree of cure (α) and temperature (T) as independent variables and the curing reaction rate (dα/dt) as the dependent variable, the model parameters were globally optimized and fitted via the particle swarm optimization (PSO) algorithm [38]. The resulting optimal parameters are listed in Table 2.

**Figure 7:**
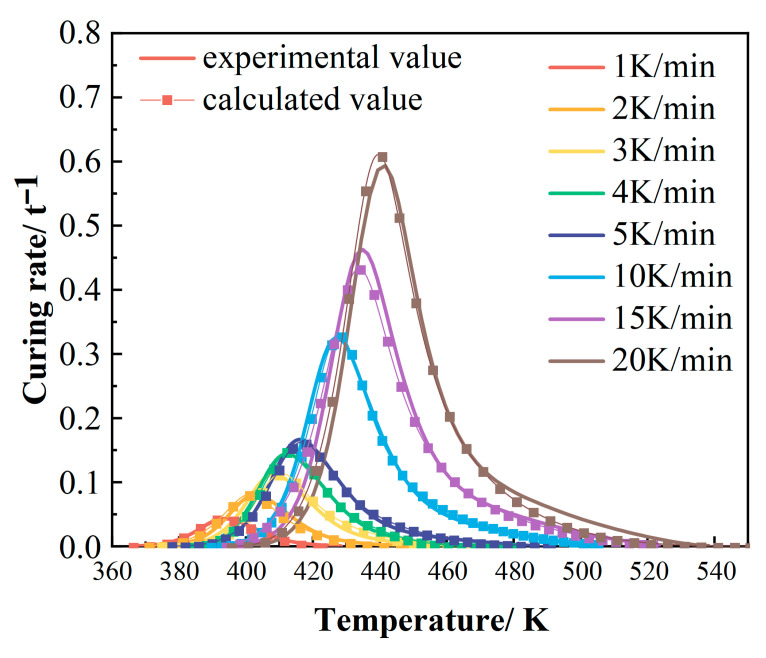
Comparison curves of experimental and calculated values of dα/dT~T at different heating rates.

To validate the compatibility of the kinetic model with experimental data, the model-predicted curing reaction rates were compared against the DSC experimental results. As shown in Figure 7, the computational results of the model demonstrate good agreement with the experimental data, indicating high consistency. This confirms that the modified Sun–Gang model accurately describes the curing process of this epoxy resin [39,40].

#### 3.1.2. Isothermal Curing Kinetics Considering the Heating Process

**Figure 8:**
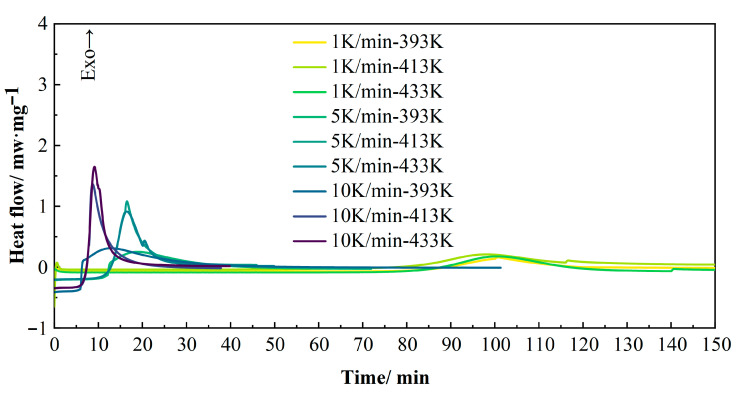
Isothermal DSC curves at different heating rates.

To optimize the curing process and minimize hold time during compression molding, isothermal DSC measurements were conducted by heating samples to temperatures of 393 K, 413 K, and 433 K at rates of 1, 5, and 10 K/min. The samples were held at each temperature for sufficient durations to ensure complete curing. This experiment aimed to evaluate the degree of cure and curing rates, thereby determining the minimum time required for optimal curing and identifying the corresponding temperature/time conditions for near-complete curing. As shown in Figure 8, the overall curing time decreased significantly with increasing heating rates. At higher heating rates (5 and 10 K/min), elevated hold temperatures resulted in intensified exothermic peaks, while the total curing completion time exhibited negligible variation.

**Figure 9:**
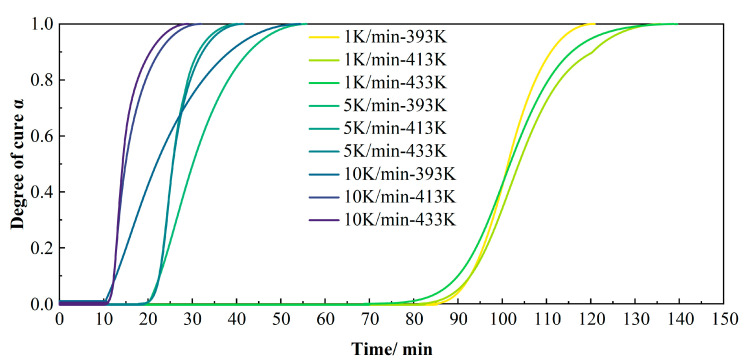
α-t curves from isothermal DSC at various heating rates (showing the degree of cure versus time).

The relationship between the degree of cure and time was further obtained from the isothermal DSC curves. Figure 9 shows the α-t curves at different heating rates. At high heating rates (5 and 10 K/min), the total curing time at 393 K was significantly longer than at 413 K and 433 K, while the difference between 413 K and 433 K was negligible. However, at low heating rates, increasing the hold temperature paradoxically extended the total curing time.

#### 3.1.3. Dynamic Versus Isothermal Viscosity Test

**Figure 10:**
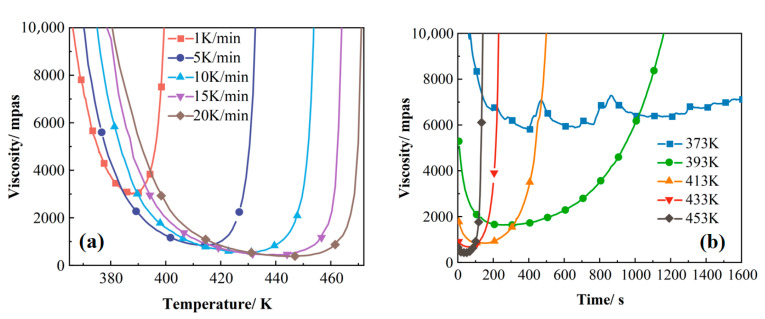
(**a**) Viscosity variation curves at different heating rates; (**b**) viscosity variation curves at different temperatures.

To further validate the curing process, rheological tests were conducted on the 2626 epoxy resin under varying heating rates and hold temperatures, with the results presented in Figure 10. Analysis indicates that the temperature range corresponding to specific rheological states (e.g., gel point) broadens with increasing heating rates, a phenomenon consistent with the peak-shift trend in non-isothermal DSC curves [41]. This fundamentally stems from intensified thermal hysteresis at higher heating rates, causing increased lag between actual sample temperature and the programmed temperature. Concurrently, the minimum viscosity decreases significantly with elevated heating rates due to synergistic control of viscosity evolution by chemical reactions (crosslink network formation) and viscous flow (enhanced molecular/segment mobility from weakened intermolecular forces at higher temperatures). At rapid heating rates, these mechanisms exhibit asymmetric competition: accelerated heating delays crosslinking, preserving more unreacted monomers/oligomers at elevated temperatures, thereby prolonging the viscous flow-dominated stage and further reducing minimum viscosity—a trend aligning with the literature reports. At fixed heating rates, increased hold temperatures substantially shorten the duration of low-viscosity states (processing window) and accelerate viscosity rise, primarily because the temperature-enhanced curing kinetics outweighs its viscosity-reducing effect. These rheological patterns (minimum viscosity variation, processing window modulation) provide critical guidance for optimizing compression molding in diverse applications, such as manufacturing large or geometrically complex components.

### 3.2. Curing Cycle Optimization Analysis

#### 3.2.1. Process Orthogonal Experiment Optimization

Curing process parameters—heating rate, hold temperature, and hold time—significantly influence the compression molding of T700/2626 epoxy composites. Consequently, this study established an orthogonal experimental design with these three factors as variables to optimize the curing cycle. A three-factor, three-level orthogonal array was designed, with factors and levels provided in Table 3. Composite mechanical properties served as evaluation metrics. The optimal curing cycle was selected based on experimental results [42]. Specific experimental designs and outcomes are presented in Table 4 (where K1, K2, and K3 denote the summed values of mechanical properties across all combinations for each corresponding level; for example, K1 represents the total ILSS value when the heating rate is 1 K/min under all associated conditions; lowercase k1, k2, and k3 indicate the average values of K1, K2, and K3, respectively; the R-value reflects the range of k-values) [43].

**Figure 11:**
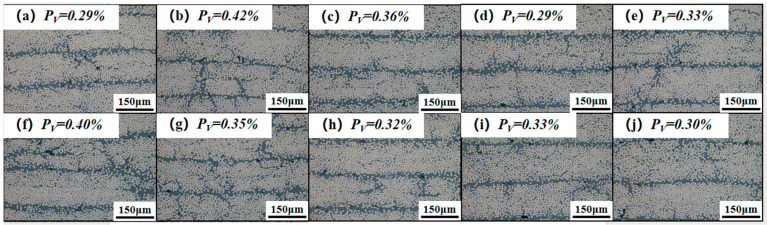
Metallographic micrograph of the composite material after compression molding: (**a**) 0-MRCC; (**b**) 1-A1B1C1; (**c**) 2-A1B2C2; (**d**) 3-A1B3C3; (**e**) 4-A2B1C2; (**f**) 5-A2B2C3; (**g**) 6-A2B3C1; (**h**) 7-A3B1C3; (**i**) 8-A3B2C1; (**j**) 9-A3B3C2.

To mitigate the influence of porosity variations in composite materials—caused by factors such as layup processes and incomplete curing—on mechanical properties, metallographic methods were employed to examine samples from different regions of the molded composites, thereby evaluating the porosity of each experimental group. Figure 11 presents metallographic micrographs of the MRCC and orthogonal experimental groups, with pore area fraction analyzed using Image Pro Plus 6.0 software. The results indicate that all porosity values fall within the range of 0.3–0.4%, well below the 1% threshold deemed acceptable in aerospace-grade manufacturing standards [44]. Experimental observations confirm minimal variation in porosity levels, with a maximum deviation of merely 0.13%, resulting in consistently negligible impact on interlaminar strength performance [45,46].

**Figure 12:**
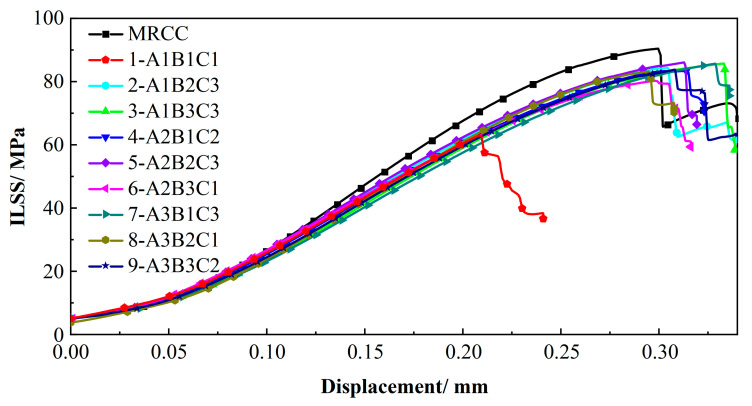
ILSS of CFRP under different curing process parameters.

Figure 12 presents the interlaminar shear strength (ILSS) results of CFRP under different curing parameters. Specimens processed with lower hold temperature and shorter hold time exhibited significantly reduced ILSS (62.88 MPa), showing a marked deviation from the 90.88 MPa achieved under MRCC conditions. In orthogonal testing, the significant variations between experimental groups indicate that process parameters significantly influence curing behavior and mechanical properties.

Based on the L9 (3^3^) orthogonal experimental design, the main effects of heating rate (A), curing temperature (B), and dwell time (C) on the interlaminar shear strength (ILSS) were quantified using range analysis (Table 5). The results show that the significance ranking of the factors is as follows: dwell time (range R = 9.92 MPa) > curing temperature (R = 8.22 MPa) > heating rate (R = 6.52 MPa). This indicates that dwell time is the dominant controlling factor for ILSS. The optimal level combination is as follows: heating rate of 10 K/min (A3), curing temperature of 413 K (B2), and dwell time of 80 min (C3). The predicted ILSS under this combination exceeds 86.15 MPa.

#### 3.2.2. Weighted Scoring for Compression Molding Process

To adapt to compression molding requirements across diverse scenarios and optimize curing cycles according to different needs, this study implemented a weighted scoring method incorporating energy consumption (electricity usage) and cycle time factors [47]. This approach enables rapid comprehensive evaluation for further optimization of curing cycles, enhancing production efficiency while reducing energy consumption. The mechanical properties, energy consumption, and compression molding time are listed in Table 6.

To eliminate dimensional discrepancies among parameters and normalize raw data to the [0,1] range, data standardization was systematically performed [48].

Positive indicators (mechanical properties):

(4) $$Z_{p}=\frac{x_{i}-x_{min}}{x_{max}-x_{min}}$$

Negative indicators (total time, energy consumption):

(5) $$Z_{N}=\frac{x_{max}-x_{i}}{x_{max}-x_{min}}$$

where Xmax and Xmin represent the maximum and minimum values of each indicator, respectively.

To scientifically quantify the relative importance of each evaluation index, this study uses the Analytic Hierarchy Process (AHP) to determine the weights. Based on expert consultation and literature analysis, an evaluation system with “process parameter optimization” as the goal layer is constructed. The criterion layer includes three core indicators: mechanical properties (C1), curing time (C2), and energy consumption (C3). The 1-9 Saaty scale was used to construct the judgment matrix (Table 7) through pairwise comparisons of criterion layer indicators [49,50,51].

The comprehensive score for each experimental group was calculated using the linear weighting method:

(6) $${Score}_{i}=\sum_{j=1}^{3}w_{j}\cdot z_{ij}$$

The results are shown in Table 8.

Considering mechanical properties as the primary factor and compression molding time and energy consumption as secondary factors, the optimal curing process route is as follows: heating at 10 K/min to 413 K and holding for 20 min. Under this curing process, compared with the MRCC, the mechanical properties are reduced by 7.35%, the compression molding time is shortened by 71.2%, and the energy consumption is reduced by 35.7%, significantly improving production efficiency. Moreover, this method can be extended to include consideration of resin flowability, thereby better formulating the curing cycle for large-sized components.

## 4. Conclusions

In this study, the curing kinetics, rheological properties, and ILSS of 2626 epoxy resin and its CFRP were systematically characterized using DSC, a rheometer, and a universal testing machine. The results show that the DSC analysis reveals the curing process is significantly controlled by the heating rate and dwell time. At higher heating rates (5, 10 K/min), when the dwell temperature is set within 393 K to 433 K, the dwell time required to reach the target degree of cure can be shortened by 15 to 35 min. The activation energy of the curing reaction calculated using the FWO method shows significant variation as the degree of cure increases. Rheological analysis monitored the viscosity evolution of the system at three temperature points of 393 K, 413 K, and 433 K. The results show that after holding for about 950 s, 450 s, and 210 s at the above temperatures, respectively, the system viscosity rises sharply. ILSS analysis, based on the orthogonal experimental design, clarifies the influence weight order of the curing process parameters (heating rate, dwell temperature, dwell time) on the interlaminar shear strength of T700/2626 epoxy resin CFRP: dwell time > dwell temperature > heating rate. Using the weighted scoring method, a new curing cycle route was developed for the T700/2626 epoxy resin system. Compared with the MRCC, the optimized process achieved significant comprehensive improvement while ensuring material performance: the interlaminar shear strength was reduced by 7.35% (meeting general product requirements), the total compression molding time was shortened by 71.2%, and the process energy consumption was reduced by 35.7%.

## Figures and Tables

**Table 1:** Thermodynamic parameters of non-isothermal DSC scans for the resin at different heating rates.

Heating Rate (K/min)	T_i_ (K)	T_p_ (K)	T_f_ (K)	∆H_R_ (J/g)
1	371.06	394.29	422.10	5.52
2	380.63	403.15	437.06	10.06
3	382.37	408.45	441.53	14.67
4	386.53	412.10	446.67	22.45
5	398.35	416.15	440.45	28.80
10	409.85	427.95	449.95	64.72
15	416.15	435.85	457.85	94.84
20	419.15	441.15	464.05	124.58

**Table 2:** Kinetic parameters and correlation coefficients of the modified Sun–Gang variable activation energy model.

A (min^−1^)	Energy (KJ/mol)	Order of Reaction
P1 = 18.25	P5 = 62.67	*m* = 0.546
P2 = 3.29	P6 = 9.58	
P3 = −1.26	P7 = 0.53	*n* = 1.192
P4 = −0.08	P8 = 3.84	

**Table 3:** Orthogonal experiment table with 3 factors, 3 levels, and 9 experimental groups.

Level	A Heating Rate (K/min)	B Temperature (K)	C Time (min)
1	1	393	20
2	5	413	50
3	10	433	80

**Table 4:** Results of the orthogonal experiment.

Experiment	Heating Rate (K/min)	Temperature (K)	Holding Time (min)	Interlaminar Shear Strength (MPa)
0-MRCC	1	393	120	90.88 ± 2.11
1-A1B1C1	1	393	20	62.28 ± 4.15
2-A1B2C2	1	413	50	84.36 ± 2.90
3-A1B3C3	1	433	80	85.71 ± 2.68
4-A2B1C2	5	393	50	83.33 ± 2.95
5-A2B2C3	5	413	80	86.15 ± 3.05
6-A2B3C1	5	433	20	80.08 ± 2.54
7-A3B1C3	10	393	80	84.45 ± 2.64
8-A3B2C1	10	413	20	84.20 ± 2.38
9-A3B3C2	10	433	50	83.26 ± 2.31

**Table 5:** Factor analysis table.

Factor	Heating Rate (K/min)	Temperature (K)	Time (min)
K1	232.35	230.06	226.56
K2	249.56	254.71	250.95
K3	251.91	249.05	256.31
k1	77.45	76.69	75.52
k2	83.19	84.90	83.65
k3	83.97	83.02	85.44
R	6.52	8.22	9.92
C > B > A			

**Table 6:** Mechanical properties, energy consumption (electricity usage), and compression molding time.

Experiment	Interlaminar Shear Strength (MPa)	Total Time (min)	Power Consumption (kWh)
MRCC	90.88 ± 2.11	250	2.10
1-A1B1C1	62.28 ± 4.15	150	1.20
2-A1B2C2	84.36 ± 2.90	210	1.65
3-A1B3C3	85.71 ± 2.68	270	2.10
4-A2B1C2	83.33 ± 2.95	100	1.50
5-A2B2C3	86.15 ± 3.05	144	1.95
6-A2B3C1	80.08 ± 2.54	98	1.50
7-A3B1C3	84.45 ± 2.64	120	1.80
8-A3B2C1	84.20 ± 2.38	72	1.35
9-A3B3C2	83.26 ± 2.31	114	1.80

**Table 7:** Criterion layer judgment matrix.

	C1	C2	C3
C1	1	3	5
C2	1/3	1	2
C3	1/5	1/2	1

The weight vector calculated using the eigenvector method is W = [0.63,0.26,0.11]. Consistency check:$\;CI=\left(\lambda_{max}-n\right)/\left(n-1\right)=0.02$, CR=CI/RI=0.03<0.1 (Passing).

**Table 8:** Calculation results and ranking of comprehensive scores.

Experiment	Mechanical Property (Score)	Total Time (Score)	Energy Consumption (Score)	Synthesis Score	Ranking
MRCC	1.00	0.10	0.00	0.66	7
1-A1B1C1	0.00	0.61	1.00	0.27	10
2-A1B2C2	0.77	0.30	0.50	0.62	8
3-A1B3C3	0.82	0.00	0.00	0.52	9
4-A2B1C2	0.74	0.86	0.67	0.76	2
5-A2B2C3	0.83	0.64	0.17	0.71	4
6-A2B3C1	0.62	0.87	0.67	0.69	6
7-A3B1C3	0.78	0.76	0.33	0.72	3
8-A3B2C1	0.77	1.00	0.83	0.83	1
9-A3B3C2	0.73	0.79	0.33	0.70	5

## Data Availability

The data presented in this study are available on request from the corresponding author.

## Abbreviations

The following abbreviations are used in this manuscript:

CFRP	Carbon fiber-reinforced polymer
MRCC	Manufacturer-recommended curing cycle
UV-FP	Ultraviolet-assisted frontal polymerization
DSC	Differential scanning calorimetry
FWO	Flynn–Wall–Ozawa
PSO	Particle swarm optimization
ILSS	Interlaminar shear strength
AHP	Analytic Hierarchy Process
